# A Comparison between Piezoelectric Devices and Conventional Rotary Instruments in Bone Harvesting in Patients with Lip and Palate Cleft: A Retrospective Study with Clinical, Radiographical, and Histological Evaluation

**DOI:** 10.1155/2018/2059464

**Published:** 2018-08-29

**Authors:** R. Rullo, A. Piccirillo, F. Femiano, L. Nastri, V. M. Festa

**Affiliations:** ^1^Associate Professor, Multidisciplinary Department of Medical-Surgical and Dental Specialties, University of Campania Luigi Vanvitelli, Naples, Italy; ^2^Resident of Oral and Maxillofacial Surgery, Multidisciplinary Department of Medical-Surgical and Dental Specialties, University of Campania Luigi Vanvitelli, Naples, Italy; ^3^Aggregate Professor, Multidisciplinary Department of Medical-Surgical and Dental Specialties, University of Campania Luigi Vanvitelli, Naples, Italy

## Abstract

**Introduction:**

Orofacial clefts are congenital malformations characterized by an incomplete shaping of structures that separate the nasal from the oral cavity and can affect the right, left, or both sides. The aim of the present study is to assess, with clinical, radiographical, and histological evaluations, the efficacy of piezoelectric devices compared to traditional rotating instruments in the bone harvesting in patients with history of cleft.

**Materials and Methods:**

We have conducted a retrospective analysis on 20 patients with a history of orofacial clefts that were operated on from February 2014 to June 2017. The patients were divided into two groups: Group R in which bone graft was harvested using a burr and Group P in which the bone graft was obtained by a piezoelectric device. After a healing period of 8 months from the grafting procedure, clinical and radiographic evaluations were performed.

**Results and Discussion:**

The use of the piezoelectric devices in bone harvesting allows a slight improvement in the final volume. This supports a faster integration into the receiving site.

**Conclusions:**

The use of piezoelectric device in patients with history of orofacial cleft that needed bone graft represents a method to be taken into consideration because it has interesting advantages.

## 1. Introduction

Orofacial clefts (OFC) are congenital malformations characterized by incomplete formation of those structures that separate the nasal and oral cavities: lip, alveolus, and hard and soft palate [1]. The incidence of this birth defect is in the range of 1 in 700 to 1 in 1000 among populations [2, 3]. It can affect either the right, the left, or both sides. If clefts are associated with any further anomaly of the body they are classified as syndromic clefts. The treatment of patients with orofacial cleft is complex and involves numerous specialists whose therapeutic phases must harmonically alternate in the rehabilitation of these patients. The restoration of the anatomical continuity of the tissues affected by the cleft, through reconstructive surgery, starts between 4 and 6 months of life and ends around two years of age; there are many different protocols used to correct this malformation. The corrective operation should restore, apart from the lip and base of nose, the continuity of the mucous tissues, leaving, however, the bone deficiency at the alveolar level. The most relevant problems defined by this deficiency are the alteration of the continuity of the maxillary arch with consequent effects on optimal development, the permanence of oronasal fistulae developed following surgery, and the lack of bone support. Previous studies have reported that the congenital absence of the permanent lateral incisor at the level of cleft is the most common result in children with cleft lip, cleft palate, or both [4, 5]. Dewinter et al. [6] found the agenesis of the lateral incisor on the cleft side in more than 50% of patients with this malformation, outside the area of cleft in 27.2% of patients, while some authors [7] detected a percentage of agenesis of 27.8 % outside the area of cleft in patients with unilateral labiocleft palate, which was significantly higher than that of the control group (no cleft) (3.6%). In case of agenesis implant-prosthetic rehabilitation offers significant advantages in terms of function, aesthetics, and quality of life and bone graft is usually needed. The bone grafting surgery is usually performed when the face become mature, at the age of eighteen, often related to the development of the maxillary canine root [8–10]. For many years one stage of surgical treatment for patients with orofacial clefts has included secondary alveolar bone grafting [11] with autologous bone [12]. This type of bone graft provides essential osteogenic cells as well as osteoinductive factors needed for bone healing and regeneration. With regard to donor site, the gold standard is bone from iliac crest [13, 14]. This requires the presence of two operative fields and an uncomfortable postoperative course also in the most conservative taking techniques. In order to perform a minimally invasive surgery, an intraoral bone harvest is increasingly used. Furthermore, the combination of autogenous bone with other biomaterials, such as platelet concentrates [15], was conducted to very good results both in bone integration and in soft tissue reparation.

Traditionally, osseous surgery is performed using hand instruments and various rotary instruments with different burs that required external copious irrigation because of the heat they produced. Over the last few decades, the rapid development of piezoelectric devices allowed overcoming the limitations of traditional instrumentation in oral bone surgery. Many authors have investigated the efficacy of piezoelectric instruments on osteotomy in terms of high precision and security of the cut, and biological respect of the tissues [16]. On the basis of the encouraging histological results of our randomized controlled clinical trial conducted on 52 cases [17], the aim of the present study is to assess the efficacy of piezoelectric devices compared to traditional rotating instruments in the bone harvesting in patients with history of cleft, from the clinical, radiographical, and histological point of view.

## 2. Materials and Methods

### 2.1. Study Population and Surgical Procedure

A retrospective review of patient charts was conducted to identify intervention of bone graft in patients with a history of unilateral complete cleft of the lip, alveolar process, and palate that were operated at the Department of Oral Surgery, University of Campania “Luigi Vanvitelli”, in Naples, Italy, from February 2014 to June 2017.

The retrospective analysis included 20 patients (the last 10 operated on by group study) in the age range from 18 to 24 years (mean age, 20.7 years), 12 boys and 8 girls, who were seen for follow-up visit 8 months after operation in order to perform an implant rehabilitation and to allow us also a clinical evaluation. Good general state of the patients' health confirmed by anaesthesiologist consultation and laboratory tests as well as prior treatment of the oral cavity were indispensable inclusion criteria. We have evaluated complete blood count (CBC), liver markers, and dosage of IgE in order to include only patients in good health without any type of disease. We have excluded smoking patients and included patients with good oral hygiene assessed at the controls. All patients underwent alveolar bone graft with mandibular bone graft by one surgeon and diagnostic, immediately postoperative, and 8 months postoperative (+/- 21 days) CBCT imaging (Carestream Dental CS 8100 3D) were taken with bite jig for data analysis. The bite jig was made at the first CBCT scan with a silicone material (polyvinyl siloxane) in order to allow a precise reproducibility in the same position of the radiographic evaluations.

The patients were divided into two groups: Group R (10 patients) and Group P (10 patients). In Group R, the bone graft was harvested using a rotating device (Medicon EG D7200 Tuttlingen–W, Germany, 220/240 V, 50 Hz, 100W) with a Stryker side cutting carbide bur n° 103 or 105 utilised at 5000-10000 rpm. In Group P, the bone graft was obtained by a piezoelectric device (Esacrom Surgysonic) with ES005T insert (thickness 0.5 mm, operative length 3.5 mm, power 50, vibra 80, water pump 100).

The local anaesthesia of all patients was carried out with 3% mepivacaine for troncular anaesthesia of mandibular nerve and 2% mepivacaine with 1: 100000 adrenaline for plexus anaesthesia.

The proposed recipient site for the graft was exposed prior to graft harvest in all cases. In this manner, the dimensions and morphology of the bony defect were measured, and minimal time elapsed between graft harvest and placement. For both groups the same surgical protocol was used because they were patients with a vertical and horizontal bone deficit given by the same kind of oral cleft.

In these patients, the receiving site presented many unfavourable elements due to the malformation. In particular, there were the remarkable dimensions of the bony defect with a thin palatine cortex inadequate for thickness as for height (class V in Cawood and Howel classification). The soft tissues characteristics due to the previous operations were sclerotic, inelastic, and with many scars. The surgeon made an intrasulcular incision from incisor to premolar with a little cut of mesial relapse. The surgeon made a cortical vivification with many microperforations, to allow the colonization of the osteoblasts and a faster graft's flourishing.

**Figure 1:**
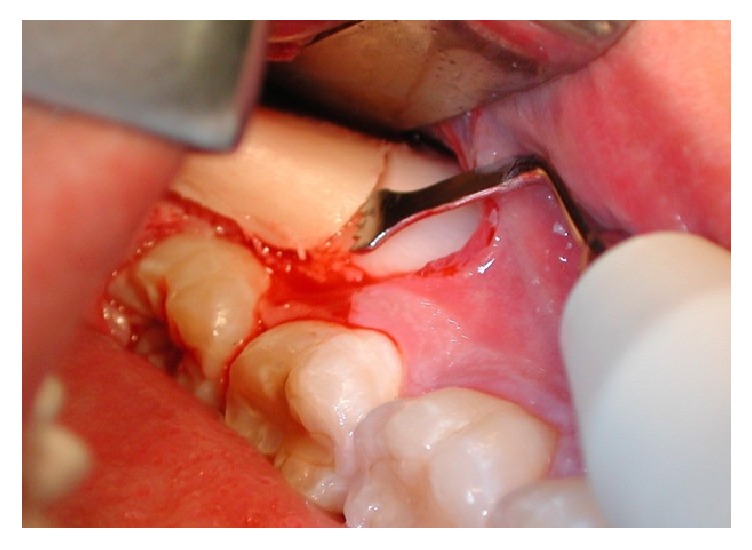
Osteotomy performed with piezoelectric device.

To access the donor area, mandibular ramus, the concavity formed by the border between the ascending ramus and the external oblique ridge was identified and used as a starting point for the mucosal incision. The incision was made medial to the external oblique ridge and extended mesially toward the buccal aspect of the second molar. Care was taken to ensure that the incision was not extended too far lingually, preventing damage to structures on the lingual aspect of the mandible. A mucoperiosteal flap was elevated, exposing the lateral aspect of the ramus. The osteotomy, started anterior to the coronoid process at a point with adequate bone thickness, was carried out with rotary instruments in Group R and with an osteotomy kit for piezoelectric surgery (Esacrom Surgysonic) in Group P (Figure 1). The cortex was cut along the anterior border of the ramus medial to the external oblique ridge. The bone block was carefully lifted to ensure that the inferior alveolar nerve was not trapped within the graft. The donor area was filled with a collagen cotton sponge for local haemostasis.

**Figure 2:**
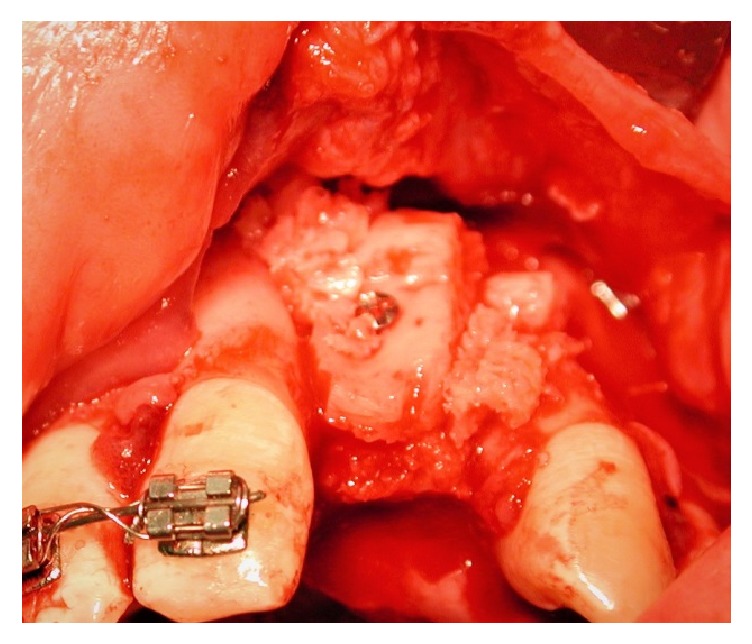
Bone graft fixed with titanium screw.

The autogenic bone, that filled the cleft fissure, was covered with lifted flaps. The incision of mucous flap for covering clefts could be moved from the lateral sides of the alveolar process. It was advised to place the bone graft in the region of the piriform aperture to provide elevation and support for the base of ala nasi on the cleft. The bone grafts, taken in a block, were then fixed with titanium osteosynthesis screws (Figure 2). Additionally, bone chips, which were harvested by using a bone scraper at the donor site as well, were packed around the bone block to fill gaps between the block graft and the recipient bone. The operation area was closed with a flap and secured with suture. Before wound closure with 4-0 nylon (polysorb) and Teflon (polytetrafluoroethylene) suture material, the entire graft was covered by a collagen membrane (Bio-Gide, Geistlich Biomaterials, Wolhusen, Switzerland). Medications were used to achieve postoperative analgesia and instructions for oral hygiene were given to patients.

After a healing period of 8 months from the grafting procedure, clinical and radiographic evaluations were performed and implants were placed.

### 2.2. Radiographic Assessment

CBCT (Carestream Dental CS 8100 3D) scans were taken before the intervention of bone graft, immediately after the operation, and after 8 months (± 21 days).

For the radiograph analysis of secondary bone grafts, a single examiner, who was previously calibrated, assessed CBCT images using a specific software.

**Figure 3:**
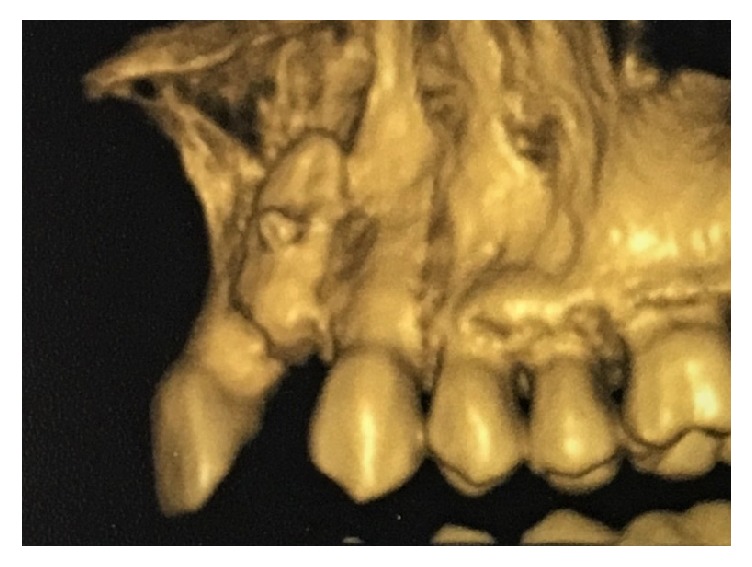
Three-dimensional image of fixed graft immediately after surgery.

All DICOM-formatted CBCT images were rendered into volumetric images using software (CS 3D Imaging Carestream, Usa) that calculated the volume of the grafted material in cubic centimetres. CBCT radiation parameters were set at 84 kV, 3.2 mA, with 15-s exposure and 1-mm scanning layer thickness (Figure 3).

### 2.3. Histological Evaluation

At the time of implant fixture application, the implant site was prepared using a bone trephine drill that allowed a bone sampling in order to carry out histological analyses and to evaluate any qualitative differences. These histologic analyses were carried out by a single calibrated operator.

### 2.4. Evaluation of Postoperative Pain, Inflammation, and Soft Tissue Healing

Postoperative pain of donor and receiving sites was assessed using a Visual Analogue Scale (VAS) [18]. The VAS consisted of a 10-cm line anchored at one end by the label “No pain” and at the other end “Worst possible pain”. The patients were given a form with three scales for the respective days after intervention (the 1^ST^, the 3^RD^, and the 7^TH^) assessed in our work.

Postoperative inflammation was estimated in donor and receiving sites. In the donor site, this evaluation was performed using two landmarks: the mandibular angle and the midline of symphysis menti. In each patient the distance between the two indicated reference points was measured in centimetres both before and after one, three, and seven days from surgery. The distance between these two points varies according to the postoperative swelling.

In the receiving site the postoperative inflammation was evaluated according to a 0 to 3 score. In this score, 0 meant absence, 1 meant slight swelling and hardness without facial planes blurring, 2 meant facial planes blurring without affectation of nasolabial folds or eyes, and 3 meant facial planes burring with affectation of nasolabial folds and eyes [19].

The assessment of soft tissue healing in donor and receiving sites was evaluated according to the score of Landry et al. [20]. This score involves the overall assessment of the tissue including epithelialization of wound boundaries, color, presence of bleeding on palpation, granulation, and suppuration.

## 3. Results

### 3.1. Radiographic Assessment

**Figure 4:**
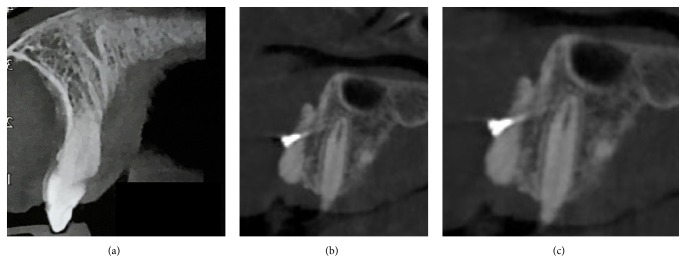
CCBCT images, before (a), immediately after (b), and after surgery (c).

For the analysis, CBCT images with a total of 20 interventions of bone graft in 20 patients were analyzed with CS 3D Imaging software immediately after and after 8 months (± 21 days) from the operation (Figure 4).

Immediately after intervention, mean postoperative values of the bone graft volume are 1.32 cm^3^  ± 0.51 for the grafts harvested with the piezoelectric devices and 1.18 cm^3^  ± 0.46 for the grafts collected with traditional instrumentation. After 8 months from operation, the mean postoperative values of the bone graft volume are 1.03± 0.51 for the graft harvested with piezoelectric devices and 0.88 ± 0.46 for the grafts collected with traditional instrumentation.

### 3.2. Histological Evaluation

**Figure 5:**
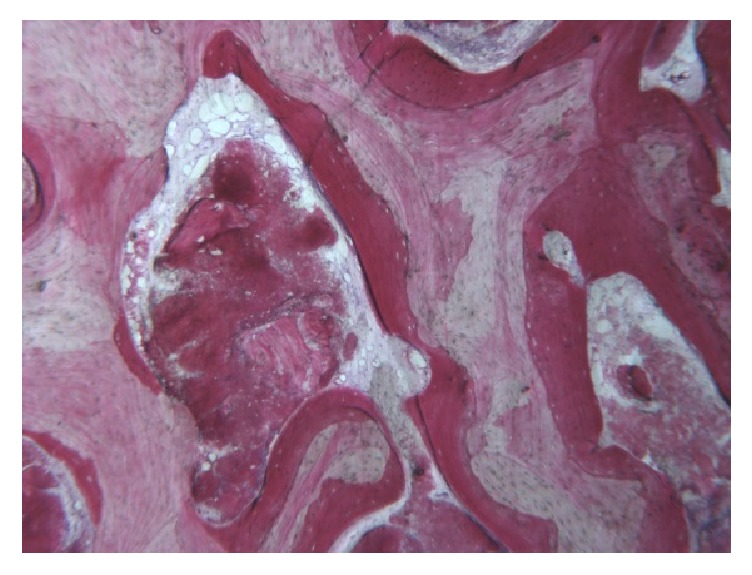
Histologic image of Group P sample.

The morphological analysis of many piezoelectric (P) device samples appeared different when compared with those taken using the rotational technique (R) (Figure 5), in particular at the point of passage between the graft and the host tissue. P samples seemed made up of well-organized and well-vascularized bone with a homogeneous appearance even at the level of the crossing point; bone samples from R were overall formed by mature bone but with less homogeneous points with the recipient site. Tissue samples from both groups displayed a normal structure, and no inflammatory infiltration was apparent.

### 3.3. Evaluation of Postoperative Pain, Inflammation, and Soft Tissue Healing

**Figure 6:**
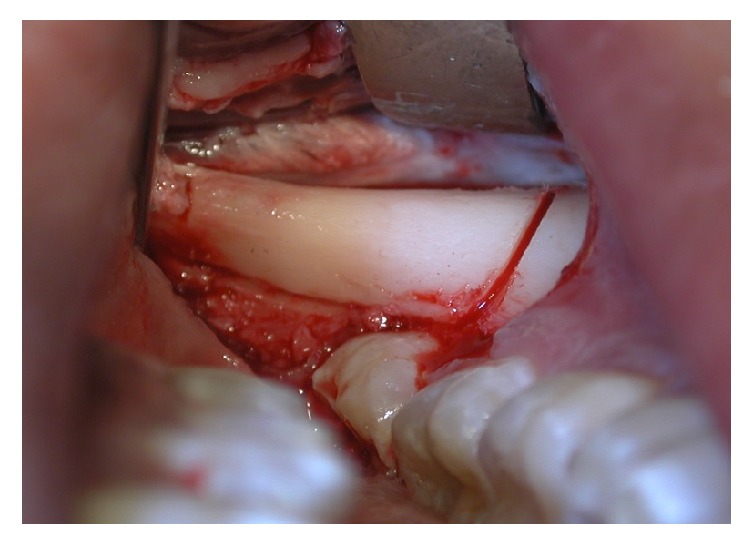
Donator site surgery.

At the donor site, the postoperative pain was slightly lower in the group treated with piezoelectric instrumentation (Group P) compared to the group treated with the traditional instrumentation (Group R) (Figure 6). In fact, at 1^ST^ day VAS average values were 6.52 ± 0.53 for Group R and 6.12 ± 0.81 for Group P; at 3^RD^ day they were 4.41 ± 0.51 for Group R and 3.18 ± 0.72 for Group P and at 7^TH^ day were 1.86 ± 0.61 for Group R and 1.43 ± 0.89 for Group P.

**Figure 7:**
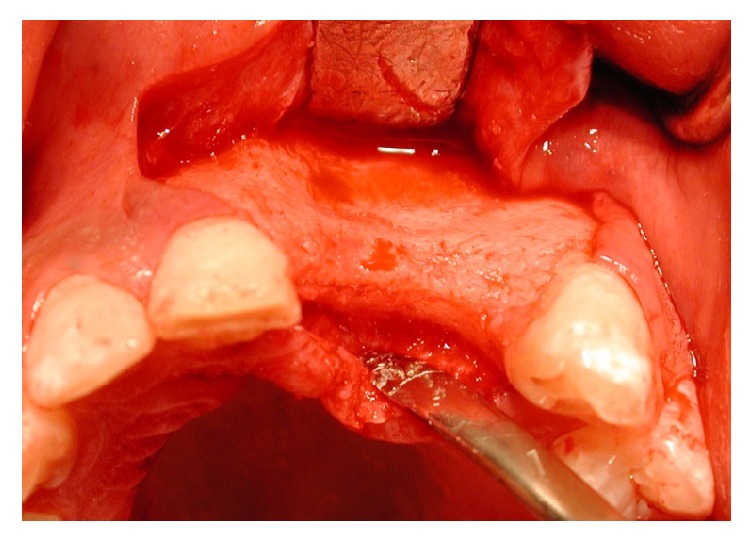
Receiving sites surgery.

**Figure 8:**
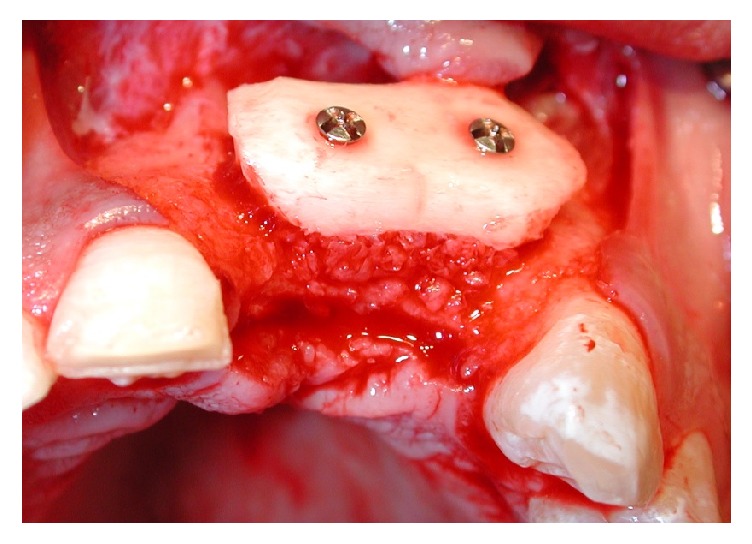
Surgery outcome.

At the receiving site (Figures 7 and 8), postoperative pain between the R and P groups was practically overlapping. Indeed, at 1^ST^ day VAS values were 4.52 ± 0.33 for Group R and 4.12 ± 0.71 for Group P, at 3^RD^ day they were 3.11 ± 0.51 for Group R and 2.18 ± 0.63 for Group P, and at 7^TH^ day they were 1.56 ± 0.51 for Group R and 1.13 ± 0.89 for Group P.

With regard to postoperative swelling at donor site, we found the following features: at 1^ST^ day in Group R the increase of distance between the two landmarks was of 23. 4%  ± 9.5% and in Group P was of 26.7%  ± 11.3%; at 3^RD^ day in Group R the increase of distance between the two landmarks was of 17.7%  ± 9.2% and in Group P was of 19.4%  ± 12.4%; at 7^TH^ day the increase of distance between the two landmarks was of 6.2%  ± 4.1% in Group R and 4.7%± in Group P.

With regard to receiving site, the postoperative inflammation evaluated according to a 0 to 3 score results in overlapping between Groups R and P. Indeed the mean score is 1.8 ± 0.5 in patients treated with traditional instruments and 1.5 ± 0.6 in patients treated with piezoelectric devices.

With regard to soft tissue healing scores, there was a statistically significant difference favouring the use of piezoelectric device. The patients of Group P showed a score included between 3.8 and 4.6, 1 week postoperatively. The patients of Group R showed a score included between 3.1 and 4.2, 1 week postoperatively.

## 4. Discussion

The restoration of a satisfactory facial aesthetics and the continuity of the alveolar process represent the main goals of in the secondary bone surgery in patients with history of cleft. The effectiveness of the operation is considered satisfactory when a sufficient volume of normally remodelled bone tissue is obtained. According to systematic reviews, success rate for implants placed in native bone is of 97% after 7 years [21, 22]. There are only a few articles in the literature focused on implantological treatment of the cleft. Landes et al. [23] demonstrated in a study that the probability of success of implants in patients with cleft is very similar to prognosis of implants inserted after traumatic tooth loss. The oral-health-related quality of life of cleft patients is similar to that of noncleft patients. Reported success rate for implants placed in the area of an alveolar cleft after bone grafting is from 80% to 90% [24, 25]: when one or more teeth are missing in the cleft area, implant placement in adulthood is the better option for function and aesthetics, contrasting bone grafting areas resorption [26, 27].

Recently, many researchers have investigated the use of allogeneic bone, artificial bone, and recombinant human bone morphogenetic protein, along with growth factors because of their ability to decrease donor-site morbidity. Both cortical and cancellous bones can be used for a bone graft, but cancellous bone is known to be better because of the cell transfer and revascularization in osteoinduction and osteoconduction. A variety of autologous, allogeneic, and xenogeneic bone materials, rhBMP, and growth factors have been used for correcting alveolar cleft. Of these, fresh autologous cancellous bone is the ideal bone graft source [28].

Iliac bone is the most commonly used bone in bone grafting because it is easy to harvest and it can provide a large amount of cancellous bone and cleft preparation can be performed at the same time. However, the disadvantage of using this bone is the presence of two operative fields with possible scarring, postoperative pain, delayed ambulation, and risk of cutaneous nerve injury [29]. This also requires the presence of two distinct surgeons and this disadvantage applies to all extraoral sites. The mandible has the same embryonic origin as the maxilla. Because it is a membranous bone and revascularization is relatively fast and resorption is low. Surgery can be performed in the same operative field and postoperative discomfort is reduced, thus reducing the length of the hospital stay.

Our school has always used intraoral sites to harvest the quantities of bone needed for cleft grafts. From a volumetric point of view, when the amount of bone required was found to be high, we utilised the autologous bone in combination with the platelet concentrates [15]. The observation in literature of the advantages of the piezoelectric devices has led in the last years to an increasing use in oral surgery [16]. This led us to apply these devices even in the grafts in patients with lip and palate cleft.

Piezoelectric bone surgery is a technique developed in the last years to overcome the limitations of traditional instrumentation in oral bone surgery by modifying and improving conventional ultrasound technology [30]. It is a meticulous and soft tissue sparing system for bone cutting based on low frequency ultrasonic microvibrations. The absence of macrovibration makes the instrument more manageable and allows greater intraoperative control with a significant increase in cutting safety in the more difficult anatomical cutting zone. Therefore, the characteristic features of bone cutting are minimal surgical trauma, a desirable control during surgery, and a rapid healing response.

Different imaging methods have been used for the assessment of bone grafts in the alveolar region, including radiographic methods [31–33], computed tomography (CT) [34, 35], and ultrasound (R. B. Lawson and M. L. Jones 1998). Rosenstein et al. [36] showed that the overall assessment of alveolar bone grafts using radiographic images was equivalent to that using CT. However, evidence suggests that CT may be a superior method to the use of conventional radiographs, as the 3-dimensional image can clearly identify bony bridge formation after grafting and the amount of bone at the receptor site, according to the bone cross-sectional image preview in the buccal-palatal direction [34, 35]. For this reason, in our work, we have evaluated CBCT images performed immediately after and after 8 months (± 21 days) from the operation. From radiographical evaluation after 8 months, the results obtained show us that in Group R, treated with traditional instrumentation, the bone volume underwent a resorption of 26% compared to the initial bone volume and 22% compared to the initial bone volume in Group P, treated with piezoelectric devices. Therefore, for our study, the piezoelectric instrumentation guarantees a better graft preservation and consequently a better volume available for the subsequent insertion of the implant fixture. Indeed, piezoelectric device allows performing definite and accurate cuts thanks to which the bone tissue is more preserved and is free of bone heat osteonecrosis. This, probably, allows seeing a radiographically lower resorption of the grafted bone.

With regard to histologic evaluation, a single blinded calibrated operator performed the analysis.

In this work we did not sacrifice the bone taken from the mandibular body at time of bone harvesting but we performed a histological evaluation of the bone after eight months from the operation, at the time of implant fixture's insertion. In many cases, the results have shown a cell maturation more advanced of the grafts treated with piezoelectric devices compared to those treated with the rotating instruments. The intervention with the piezoelectric instruments allows having, on average, an amount of graft greater due to a lower bone resorption of the bone applied in the site of the cleft. We think that the lack of bone heat osteonecrosis of bone graft allows a faster integration of graft in receiving site.

In theory harvesting bone from the mandibular body and ramus region can cause severe complications, like fracture of the mandible [37] or sensorial disturbances of the lingual or mandibular nerve [38]. However, this cannot be confirmed with this study in which none of these severe complications occurred in 20 patients treated. However, a disadvantage of grafts from the mandibular region remains. Only a confined amount of bone can be harvested from this donor site. It has been described that the volume is half of what can be achieved from the mandibular symphysis [39]. The limits of the retromolar area are usually determined by the reduced clinical access and the limited view. We had evaluated the postoperative state in a simple manner with VAS scale, using landmarks and with score of Landry et al. 1988. From a symptomatological point of view, the major disorder reported by the patients treated in our work is at the donor site, at the level of the body of the jaw but, in this area, the healing is faster than the receiving site. Indeed, at the site of the graft the surgical wound heals more slowly and with greater risks of complications. The postoperative course is instead superimposable between the two methods used. With regard to our experience, this result basically depends on the influence that the duration of the intervention has on postoperative inflammation. Indeed, the cuts performed with rotary instruments were performed in reduced times; therefore the patients explain less swelling compared to patients treated with piezoelectric devices.

## 5. Conclusions

The use of the piezoelectric devices allows a slight improvement in the final volume. We hypothesize that this may be due to the lower necrosis of the bone block taken with this method, as found in the histological examinations. This leads to faster integration into the receiving site. Although it is objective that the piezoelectric device is less damaging to the tissue, as regards the inflammation, we have found an overlapping inflammation both in the sampling site and in the grafting site. This is because we believe that the inflammation depends more on the duration of the intervention rather than on the methodology, in accord with our study done on the included third molars [17].

According to the results of present study, it can be expected that the use of piezoelectric device in bone harvesting represents a method to be taken into consideration because it has interesting advantages in terms of greater postoperative comfort, respect for noble anatomical structures, and faster integration of the graft.

From our point of view an interesting evaluation that allows comparing the characteristics of the tissues taken with the two different techniques illustrated would be to carry out a randomized, and not a retrospective, clinical study which allows obtaining results on a larger patient sample.

## Data Availability

The data used to support the findings of this study are available from the corresponding author upon request.
