# Corrigendum to “A Comparison between Piezoelectric Devices and Conventional Rotary Instruments in Bone Harvesting in Patients with Lip and Palate Cleft: A Retrospective Study with Clinical, Radiographical, and Histological Evaluation”

**DOI:** 10.1155/2018/6751495

**Published:** 2018-10-22

**Authors:** R. Rullo, A. Piccirillo, F. Femiano, L. Nastri, V. M. Festa

**Affiliations:** ^1^Associate Professor, Multidisciplinary Department of Medical-Surgical and Dental Specialties, University of Campania Luigi Vanvitelli, Naples, Italy; ^2^Resident of Oral and Maxillofacial Surgery, Multidisciplinary Department of Medical-Surgical and Dental Specialties, University of Campania Luigi Vanvitelli, Naples, Italy; ^3^Aggregate Professor, Multidisciplinary Department of Medical-Surgical and Dental Specialties, University of Campania Luigi Vanvitelli, Naples, Italy

In the article titled “A Comparison between Piezoelectric Devices and Conventional Rotary Instruments in Bone Harvesting in Patients with Lip and Palate Cleft: A Retrospective Study with Clinical, Radiographical, and Histological Evaluation” [1], an acknowledgment should be added as follows:

The authors would like to acknowledge the Valere Project of the University of Campania Luigi Vanvitelli for the support to publish this article.
